# Obstructive Sleep Apnea and Obesity Are Associated with Hypertension in a Particular Pattern: A Retrospective Study

**DOI:** 10.3390/healthcare11030402

**Published:** 2023-01-31

**Authors:** Yunyan Xia, Caihong Liang, Junxin Kang, Kai You, Yuanping Xiong

**Affiliations:** 1 Department of Otorhinolaryngology–Head and Neck Surgery, First Affiliated Hospital of Nanchang University, Nanchang 330006, China; 2 Department of Anesthesiology, First Affiliated Hospital of Nanchang University, Nanchang 330006, China

**Keywords:** obstructive sleep apnea, obesity, hypertension, interaction

## Abstract

Obstructive sleep apnea (OSA) and obesity can increase the risk of hypertension, but the combined effects of these two conditions on hypertension are not yet known. We collected the basic characteristics, sleep parameters, and glucose levels of subjects with a polysomnography test and divided them into four groups, according to whether they had severe OSA and obesity or not. The main effects of severe OSA and obesity and the interactions of the two on systolic blood pressure (SBP) and diastolic blood pressure (DBP) levels were detected using analysis of covariance. The association between obesity and severe OSA and abnormal blood pressure and their combined effects were detected with logistic regression. In total, 686 subjects were included. After adjusting for multiple confounding factors, the strong main effects of obesity and severe OSA were detected in the SBP and DBP levels, with no combined effects from the two conditions on SBP or DBP. Obesity was independently associated with the presence of hyper-systolic blood pressure (hyper-SBP) and hypertension, and severe OSA was independently associated with the presence of hyper diastolic blood pressure (hyper-DBP) and hypertension. No effects of the interaction between severe OSA and obesity on the presence of abnormal blood pressure were observed. Both severe OSA and obesity were associated with hypertension, while obesity was closely associated with hyper-SBP, and severe OSA was associated with hyper-DBP. No effects of the interaction between these two on hypertension were observed.

## 1. Introduction

Obstructive sleep apnea (OSA) is one of the most common sleep disorders, and the prevalence of moderate-to-severe OSA in females and males has been shown to reach up to 23.4% and 49.7%, respectively [1]. Positive associations between OSA and hypertension have been established by many studies. The prevalence of hypertension in mild, moderate, and severe OSA is 59%, 62%, and 67%, respectively, as reported in the Sleep Heart Health study [2]. Furthermore, after adjusting for multiple confounding factors, the OSA severity index and the apnea–hypopnea index (AHI) are significant independent predictors of both systolic blood pressure (SBP) and diastolic blood pressure (DBP) [3]. Meanwhile, SBP and DBP have been shown to increase in line with increasing OSA severity [4]. In addition, our previous study also demonstrated that subjects with more severe OSA have significantly higher SBP and DBP levels and are more likely to have hypertension than those with less severe OSA [5].

Obstructive sleep apnea and obesity often coexist. The prevalence of OSA is continuously increasing with the epidemic of obesity. One previous study showed that in a non-obese population, the prevalence rates of OSA were 3.0% in males and 0.7% in females, while in an obese population, the prevalence rates increased to 12.1% in males and 7.0% in females [6]. In the Sleep Heart Health Study, a weight gain of 10 kg over a 5-year period conferred a 5.2- and 2.5-fold increase in the likelihood of increasing the AHI by 15 events per hour in men and women, respectively [7]. Furthermore, OSA is often accompanied by obesity. It has been reported that most adult patients with OSA are more likely to be centrally obese and have more visceral fat accumulation than those without OSA [8,9]. Similar to OSA, obesity is an important risk factor for hypertension. The Framingham Heart Study and the HYDRA study exhibited strong correlations between obesity and poor blood pressure control [10], and obesity was identified as one of the three main independent risk factors associated with poor blood pressure control [11]. Furthermore, interventions to reduce adiposity and avoid excess weight may have large effects on the development of hypertension at an individual and population level [12].

Thus, both obesity and OSA can increase the risk of hypertension. Previous studies have shown that OSA and obesity promote each other’s development [13,14,15] and—inspired by our previous studies demonstrating the combined effects of OSA and obesity on dyslipidemia and cognitive function [16,17] and one study showing the modification effect of obesity on the treatment of positive airway pressure and cardiac function [18]—we postulated that OSA and obesity exert a combined effect on blood pressure and hypertension. However, the interaction between OSA and obesity in hypertension in an adult population has not yet been confirmed. Thus, we performed this study to investigate the interrelationships between obesity, OSA, and hypertension.

## 2. Materials and Methods

### 2.1. Subjects

**Figure 1:**
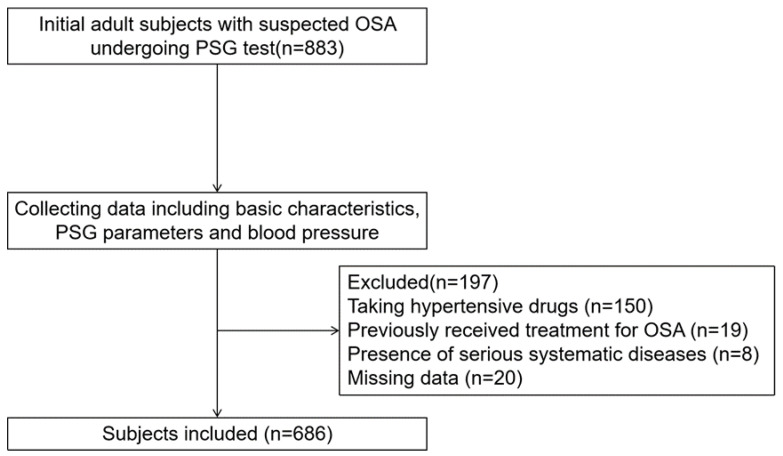
Enrollment flow chart of subjects. Abbreviations: OSA, obstructive sleep apnea; PSG, polysomnography.

Adult patients with suspected OSA with symptoms of snoring receiving polysomnography (PSG) tests between January 2014 and August 2022 at the First Affiliated Hospital of Nanchang University were included. The exclusion criteria included taking hypertensive drugs previously (*n* = 150); previous OSA treatment (*n* = 19); serious systemic diseases (e.g., heart failure) (*n* = 8); and missing data (*n* = 20). A total of 686 patients with complete data were included (See in Figure 1).

The study was approved by the Internal Review Board of the Institutional Ethics Committee of the First Affiliated Hospital of Nanchang University (Approval No. 2020-139) and was conducted in accordance with all relevant tenets of the Declaration of Helsinki. The informed consent from the study participants prior to study commencement was not required by the ethics committee, as this was a retrospective study.

### 2.2. Basic Characteristics

All included patients provided a comprehensive medical history. The medical history included gender, age, whether the patients had a history of hypertension and/or diabetes, whether they were on drugs to treat hypertension and/or diabetes, and their smoking and alcohol consumption status. A history of hypertension was defined as patients diagnosed with hypertension by a doctor before the study or patients who measured blood pressure three times on different days at home and found systolic blood pressure (SBP) ≥140 mmHg and (or) diastolic blood pressure (DBP) ≥90 mmHg without taking antihypertensive drugs in accordance with the Guidelines for Prevention and Treatment of Hypertension in China [19]. Patients were divided into non-smokers, current smokers, and ex-smokers; we also recorded the mean number of cigarettes smoked per day, smoking duration (years), and number of years since quitting (for ex-smokers). Non-smokers were defined as those who had never smoked cigarettes. Current smokers were those who self-reported cigarette use for at least 12 months. Ex-smokers were former smokers who had given up after smoking for at least 12 months and abstained for at least 12 months. Those who had been smokers in the past were defined as smokers in this study. Patients were also classified, according to alcohol consumption status, as non-drinkers, current drinkers, or ex-drinkers. Those who had been drinkers in the past were defined as drinkers in this study. Blood tests were also performed, including fasting glucose levels.

### 2.3. Anthropometric Measurements

The weights and heights of subjects were collected. The body mass index (BMI) was calculated as weight (kg)/height (m)^2^. According to the Guidelines for Prevention and Control of Overweight and Obesity in Chinese Adults, obesity was defined as BMI  ≥  28 kg/m^2^ [20].

### 2.4. Overnight PSG Parameters

All subjects received II-level PSG tests, which were conducted on wards. Electroencephalogram (EEG), electrooculogram (EOG), electrocardiogram (ECG), electromyogram (EMG), nasal and oral airflow, thoracic and abdominal respiratory effort, pulse oximetry, posture, and snoring data were obtained. According to AASM criteria, apnea referred to the absence of or significant reduction in nasal and oral airflow during sleep (at least 90% reduction from baseline) for at least 10 s or more. Hypopnea was defined as a nasal and oral airflow amplitude decrease of 30% or more compared with waking, with blood oxygen saturation decreasing by 4% or above for more than 10 s or a nasal and oral airflow amplitude decrease of ≥50% with blood oxygen saturation decreasing by 3% or above for a duration of 10 s or more. The apnea–hypopnea index (AHI) was defined as the number of apnea and hypopnea events per hour during sleep. The oxygen desaturation index (ODI) was defined as the number of times per hour of sleep that the blood oxygen level dropped by ≥4% from the baseline. The lowest oxygen saturation value during sleep was referred to as the LSpO_2_. According to AASM criteria, the AHI was categorized as <30 and ≥30 events per hour and represented non-severe OSA and severe OSA, respectively [21].

### 2.5. Blood Pressure

Blood pressure was measured three times with an automatic sphygmomanometer after at least 5 min of rest in a sitting position, in accordance with the American Society of Hypertension guidelines, and the mean values were calculated. According to the Guidelines for Prevention and Treatment of Hypertension in China, hypertension was defined as abnormal SBP level (≥140 mmHg), abnormal DBP level (≥90 mmHg), or both [19]. Hyper-systolic blood pressure (hyper-SBP) was defined as SBP ≥ 140 mmHg without consideration of DBP level. Similarly, hyper-diastolic blood pressure (hyper-DBP) was defined as DBP ≥ 90 mmHg without consideration of SBP level.

### 2.6. Statistical Analysis

Continuous data are presented as medians (with interquartile range). Categorical data are presented as numbers (%). Subjects were divided into four groups according to obesity and severe OSA, including a non-obese-with-non-severe-OSA group, a non-obese-with-severe-OSA group, an obese-with-non-severe-OSA group, and an obese-with severe-OSA group. The differences in basic characteristics, PSG parameters, and blood pressure levels between the four groups were examined using ANOVA (analysis of variance) tests, and post hoc tests were performed with Dunnett’s T3 tests. Differences in categorial data between the groups were examined with chi-square tests or Fisher’s exact tests, and post hoc tests were performed with z-tests. Analyses of covariance (ANCOVA) were used to determine the main effects of interaction between obesity and severe OSA on blood pressure levels while adjusting for multiple confounding factors, including gender, age, glucose level, drinking, and smoking. We used binary logistic regression analyses to detect the risk factors for hyper-SBP, hyper-DBP, and hypertension and the effects of the interactions between severe OSA and obesity on the presence of hypertension. All statistical analyses were performed using SPSS Statistics 21.0 software (IBM Corp., Armonk, NY, USA). Values of *p* < 0.05 were taken to indicate statistical significance.

## 3. Results

In total, 686 subjects were enrolled, including 232 non-obese subjects with non-severe OSA (Group a), 201 non-obese subjects with severe OSA (Group b), 61 obese subjects with non-severe OSA (Group c), and 192 obese subjects with severe OSA (Group d).

### 3.1. Basic Characteristics and PSG Parameters Stratified by Obesity and Severe OSA

In both the non-obese and the obese categories, those with severe OSA had a significantly higher percentage of males and significantly higher AHI and ODI and lower LSpO_2_ than those with non-severe OSA (all *p* < 0.05). In the non-obese category, those with severe OSA had a higher BMI than those with non-severe OSA (*p* = 0.005). No significant differences in age, glucose level, percentage of smokers, drinkers, and diabetes between those with severe OSA and those with non-severe OSA were seen in either the non-obese or the obese category (all *p* > 0.05; see Table 1).

### 3.2. Blood Pressure Levels and Percentages of Abnormal Blood Pressure Stratified by Obesity and Severe OSA

**Figure 2:**
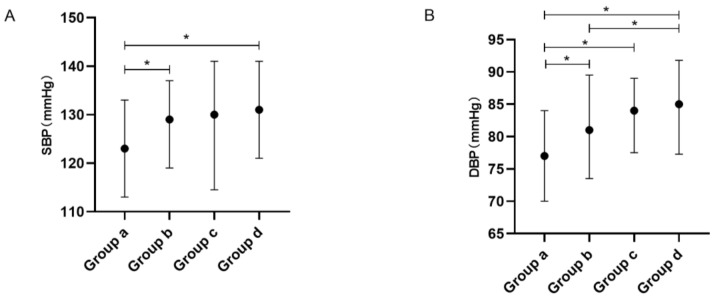
Blood pressure levels stratified by obesity and severe OSA. Footnote: Group a, non-obese with non-severe OSA group; Group b, non-obese with severe OSA group; Group c, obese with non-severe OSA group; Group d, obese with severe OSA group; subfigure (**A**), comparison of SBP level among the four gruops; subfigure (**B**), comparison of DBP level among the four groups; *, *p* < 0.05. Abbreviations: OSA, obstructive sleep apnea; SBP, systolic blood pressure; DBP, diastolic blood pressure.

For overall comparison, significant differences in SBP and DBP were seen between the four groups (all p^1^ < 0.001). The post hoc tests showed that the non-obese subjects with non-severe OSA had significantly lower SBP levels than those who were non-obese with severe OSA and those who were obese with severe OSA and had significantly lower DBP levels than the other three groups (all *p* < 0.05). The non-obese subjects with severe OSA had significantly lower DBP levels than those who were obese with severe OSA (see Table 2 and Figure 2).

**Figure 3:**
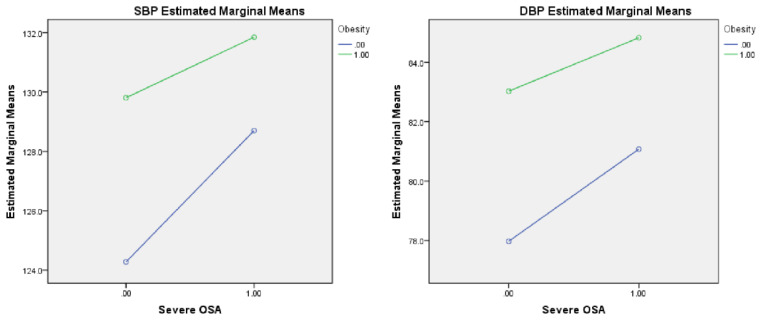
Interaction plot between obesity and severe OSA on blood pressure. Footnote: The figure shows no significant interaction between obesity and severe OSA in SBP and DBP. Abbreviations: OSA, obstructive sleep apnea; SBP, systolic blood pressure; DBP, diastolic blood pressure.

After adjusting for gender, age, glucose level, smoking, and drinking, the strong main effects of severe OSA were detected in SBP (F = 5.416, *p* = 0.020) and DBP (F = 5.686, *p* = 0.017). Furthermore, after adjusting for the multiple confounding factors described above, the strong main effects of obesity were detected in SBP (F = 9.652, *p* = 0.002) and DBP (F = 18.144, *p* < 0.001; see Table 2). No effects from interactions between severe OSA and obesity on SBP (F = 0.764, *p* = 0.382) or DBP (F = 0.418, *p* = 0.518) were found (see Table 2 and Figure 3).

**Figure 4:**
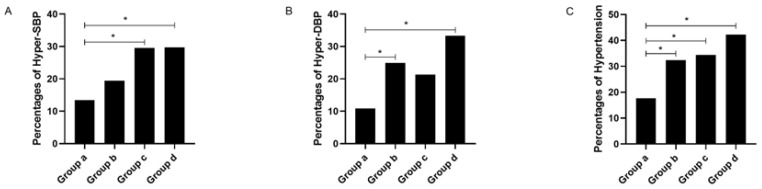
Percentages of abnormal blood pressure stratified by obesity and severe OSA. Footnote: Group a, non-obese with non-severe OSA group; Group b, non-obese with severe OSA group; Group c, obese with non-severe OSA group; Group d, obese with severe OSA group; subfigure (**A**), comparison of percentages of hyper-SBP among the four gruops; subfigure (**B**), comparison of percentages of hyper-DBP among the four groups; subfigure (**C**), comparison of percentages of hypertension among the four groups; *, *p* < 0.05. Abbreviations: OSA, obstructive sleep apnea; SBP, systolic blood pressure; DBP, diastolic blood pressure.

In the overall comparison, significant differences in the percentages of hyper-SBP, hyper-DBP, and hypertension were seen between the four groups (all p^1^ < 0.001). The post hoc test showed that the non-obese subjects with non-severe OSA had a significantly lower percentage of hyper-SBP than the obese subjects with non-severe OSA and the obese subjects with severe OSA, a significantly lower percentage of hyper-DBP than the non-obese subjects with severe OSA and the obese subjects with severe OSA, and a significantly lower percentage of hypertension than the other three groups (all *p* < 0.05; see Table 3 and Figure 4).

### 3.3. Associations between Obesity and Severe OSA and Abnormal Blood Pressure

The logistic regression models showed that, after adjusting for gender, age, glucose level, drinking, and smoking, obesity was independently associated with the presence of hyper-SBP (OR (95%CI) = 2.695 (1.335, 5.440), *p* = 0.006) and hypertension (OR (95%CI) = 2.365 (1.233, 4.535), *p* = 0.010). After adjusting for multiple confounding factors, severe OSA was independently associated with the presence of hyper-DBP (OR (95%CI) = 2.702 (1.585, 4.607), *p* < 0.001) and hypertension (OR (95%CI) = 2.285 (1.438, 3.631), *p* < 0.010). No effects of the interaction between severe OSA and obesity on hyper-SBP (OR (95%CI) = 0.670 (0.288, 1.558), *p* = 0.352), hyper-DBP (OR (95%CI) = 0.694 (0.290, 1.659), *p* = 0.412), or hypertension (OR (95%CI) = 0.636 (0.294, 1.373), *p* = 0.249) were observed (see Table 4).

## 4. Discussion

In this study, we found significant differences in blood pressure levels, including SBP and DBP, along with significant differences in percentages of abnormal blood pressure, including hyper-SBP, hyper-DBP, and hypertension, between the four groups stratified by severe OSA and obesity. After adjusting for multiple confounding factors, the strong main effects of both obesity and severe OSA were detected in SBP and DBP. No effects of the interactions between severe OSA and obesity on SBP or DBP were observed. The logistic regression models showed that obesity was independently associated with the presence of hyper-SBP and hypertension, and severe OSA was independently associated with the presence of hyper-DBP and hypertension. No effects of the interaction between severe OSA and obesity on the presence of hyper-SBP, hyper-DBP, or hypertension were observed.

Previous studies have shown that obesity and OSA are related to microvascular dysfunction and that the latter condition is associated with arterial hypertension and might be a target for specific treatments [22,23]. Thus, we closely explored the relationships between OSA, obesity, and hypertension. Obstructive sleep apnea has long been identified as an important risk factor in the development of hypertension, especially resistant hypertension. Both clinical and rodent studies have shown that OSA is associated with a rise in systemic blood pressure. A combination of severe mechanisms, mainly intermittent hypoxia and microarousals, is thought to result in OSA-related hypertension [24]. In addition to this, we found that severe OSA was associated with hyper-DBP but not hyper-SBP. Some other studies have also mentioned this particular pattern in the relationship between OSA severity and blood pressure. Hu et al. found that DBP, but not SBP, was independently associated with AHI, ODI, and the arousal index after adjustment for obesity and other risk factors [25]. Tryfon et al. found that normotensive OSA patients develop DBP elevation, but not SBP elevation, at an earlier stage during exercise compared with normal subjects [26]. Wu et al. showed that repetitive arousals (RAs) result in the elevation of DBP without significant changes in SBP [27]. The elevation of DBP is typically considered to increase peripheral resistance, which is mainly produced by small arterioles, whereas resistance in the conductance vessels is predominantly responsible for SBP elevation. A rise in DBP without a rise in SBP suggests a more prominent impact from OSA on the peripheral vessels rather than on the moderate-to-large vessels [24].

Several studies have shown a clear positive association between blood pressure levels and weight gain. Data from NHANES indicate that the prevalence of hypertension among obese individuals is 42.5% compared with 15.3% for non-overweight individuals [28]. The Framingham Heart Study found that age-adjusted relative risk (RR) for new hypertension was strongly associated with overweight status [12]. In addition, weight loss is important for the prevention and treatment of hypertension. A meta-analysis showed that with each kilogram of weight loss, systolic blood pressure was reduced by −1.05 mmHg, and diastolic blood pressure was reduced by −0.92 mmHg [29]. The pathogenesis of obesity-related hypertension includes insulin resistance, increased leptin levels, increased SNS activity, increased RAAS activity, and impaired salt sensitivity [30]. In our study, we found a significant association between obesity and hyper-SBP but not hyper-DBP. However, most previous studies showed that obesity not only influences the small arterioles but also affects the conductance vessels [30,31]. We used BMI as an index of obesity, but another obesity index, the waist-to-hip ratio, might be more relevant to cardiovascular risks. This might explain the lack of association between obesity and hyper-DBP. More studies are needed to further clarify this particular pattern.

Obesity has been reported to exacerbate OSA by reducing the size of the upper-airway lumen by increasing fat accumulation [13], decreasing chest-wall compliance, and increasing airway resistance [14]. Furthermore, intermittent hypoxia has been shown to cause obesity [15]. Thus, obesity and OSA have been shown to exacerbate each other’s occurrence and development. We postulated that the interaction between OSA and obesity might affect blood pressure and the presence of abnormal blood pressure. However, unlike the effect of the interaction of obesity and OSA on specific dyslipidemia and cognitive function found in previous studies [16,17], no effects of the interaction between severe OSA and obesity on blood pressure levels and the presence of abnormal blood pressure were observed. In this study, we found that severe OSA was closely related to hyper-DBP, while obesity was closely related to hyper-SBP; this might partially explain the lack of interaction. More largescale clinical studies and rodent studies are needed to further analyze the effects of the interaction between these two conditions on blood pressure and hypertension and determine the underlying mechanism.

This study had some limitations. First, the retrospective study design found no causality. Furthermore, the integrity of all the data, such as some detailed PSG parameters, including sleep stages and the arousal index (representing sleep fragmentation), could not be guaranteed. Second, since the population was hospital-based, the conclusions cannot be extended to other populations. Third, we calculated BMI as an index of obesity, but another obesity index, the waist-to-hip ratio, might be more relevant to cardiovascular risks. Despite these limitations, the sleep data based on PSG and objective measurements increased the credibility of our results.

## 5. Conclusions

In conclusion, we found strong independent main effects for both severe OSA and obesity on blood pressure and did not find effects due to interactions between these two conditions on blood pressure levels. Both obesity and severe OSA were independently associated with hypertension, while obesity was closely associated with hyper-SBP, and severe OSA was associated with hyper-DBP. No effects of the interaction between obesity and severe OSA on the presence of abnormal blood pressure were observed.

## Figures and Tables

**Table 1:** Basic characteristics and PSG parameters stratified by obesity and severe OSA.

	Whole Subjects (N = 686)	Non-Obese Category (N = 433)			Obese Category (N = 253)		
		Non-Severe OSA Group (Group a, N = 232)	Severe OSA Group (Group b, N = 201)	*p*	Non-Severe OSA Group (Group c, N = 61)	Severe OSA Group (Group d, N = 192)	*p*
Basic characteristics							
Male, N (%)	589 (85.9)	177 (76.3)	182 (90.5)	<0.001	50 (82.0)	180 (93.8)	0.005
Age	42.0 (32.0, 51.0)	46.0 (33.3, 54.0)	43.0 (32.0, 51.0)	0.281	44.0 (30.5, 51.0)	37.0 (30.0, 45.0)	0.320
BMI	26.9 (24.6, 29.4)	24.8 (22.9, 26.4)	25.4 (24.2, 26.9)	0.005	29.8 (29.0, 31.3)	30.5 (29.3, 32.7)	0.409
Smoking, N (%)	257 (37.5)	72 (31.0)	68 (33.8)	0.535	28 (45.9)	89 (46.4)	0.951
Drinking, N (%)	106 (15.5)	31 (13.4)	32 (15.9)	0.451	11 (18.0)	32 (16.7)	0.805
Diabetes, N (%)	26 (3.8)	7 (3.0)	5 (2.5)	0.738	3 (4.9)	11 (5.7)	1.000
Glucose	5.17 (4.68, 5.76)	5.13 (4.63, 5.56)	5.06 (4.59, 5.57)	0.992	5.17 (4.62, 6.21)	5.39 (4.85, 6.02)	1.000
PSG parameters							
AHI	37.1 (16.7, 61.7)	10.9 (4.7, 21.3)	56.6 (41.4, 65.7)	<0.001	16.9 (6.1, 22.0)	64.8 (48.7, 64.8)	<0.001
ODI	35.4 (14.4, 63.9)	10.0 (3.6, 20.7)	53.9 (40.1, 67.7)	<0.001	16.0 (6.5, 26.3)	67.3 (49.9, 84.7)	<0.001
LSpO_2_	77.0 (65.0, 85.0)	86.0 (81.0, 90.0)	71.0 (61.0, 78.0)	<0.001	84.0 (79.0, 88.0)	65.5 (54.3, 73.0)	<0.001

ANOVA tests were used to detect the differences in continuous data among the four groups, and post hoc tests were performed with Dunnett’s T3 tests. Chi-square tests or Fisher’s exact tests were used to detect the differences in categorial data among the four groups, and post hoc tests were performed with z-tests. Abbreviations: OSA, obstructive sleep apnea; AHI, apnea–hypopnea index; ODI, oxygen desaturation index; LSpO_2_, lowest oxygen saturation value during sleep.

**Table 2:** Blood pressure levels stratified by obesity and severe OSA.

	Whole Subjects (N = 686)	Non-Obese Category (N = 433)		Obese Category (N = 253)		p^1^	Main Effect, p^2^		Interaction, p^3^
		Non-Severe OSA Group (Group a, N = 232)	Severe OSA Group (Group b, N = 201)	Non-Severe OSA Group (Group c, N = 61)	Severe OSA Group (Group d, N = 192)		Obesity	Severe OSA	
Blood pressure levels									
SBP	128.0 (117.0, 138.0)	123.0 (113.0, 133.0) ^ab, ad^	129.0 (119.0, 137.0)	130.0 (114.5, 141.0)	131.0 (121.0, 141.0)	<0.001	0.002	0.020	0.382
DBP	81.0 (73.0, 89.0)	77.0 (70.0, 84.0) ^ab,ac,ad^	81.0 (73.5, 89.5) ^bd^	84.0 (77.5, 89.0)	85.0 (77.3, 91.8)	<0.001	<0.001	0.017	0.518

ANOVA tests were used to detect the differences in blood pressure among the four groups, and post hoc tests were performed with Dunnett’s T3 tests. ANCOVA tests were used to detect the main effect and interaction effect of obesity and severe OSA on blood pressure while adjusting for confounding factors, including gender, age, smoking, drinking, and glucose levels. ^ab^, *p* < 0.05 for the comparison between the non-obese with non-severe OSA group and the non-obese with severe OSA group. ^ac^, *p* < 0.05 for the comparison between the non-obese with non-severe OSA group and the obese with non-severe OSA group. ^ad^, *p* < 0.05 for the comparison the between non-obese with non-severe OSA group and the obese with severe OSA group. ^bd^, *p* < 0.05 for the comparison between the non-obese with severe OSA group and the obese with severe OSA group. p^1^, overall comparison between the four groups. p^2^, *p*-value after adjusting for the factors of gender, age, glucose level, smoking, and drinking. p^3^, *p*-value for interaction effect adjusting for the factors of gender, age, glucose level, smoking, and drinking. Abbreviations: OSA, obstructive sleep apnea; SBP, systolic blood pressure; DBP, diastolic blood pressure.

**Table 3:** Percentages of abnormal blood pressure stratified by obesity and severe OSA.

	Whole Subjects (N = 686)	Non-obese Category (N = 433)		Obese Category (N = 253)		p^1^
		Non-Severe OSA Group (Group a, N = 232)	Severe OSA Group (Group b, N = 201)	Non-Severe OSA Group (Group c, N = 61)	Severe OSA Group (Group d, N = 192)	
Percentages of abnormal blood pressure						
Hyper-SBP, N (%)	145 (21.1)	31 (13.4) ^ac,ad^	39 (19.4)	18 (29.5)	57 (29.7)	<0.001
Hyper-DBP, N (%)	152 (22.2)	25 (10.8) ^ab,ad^	50 (24.9)	13 (21.3)	64 (33.3)	<0.001
Hypertension, N (%)	208 (30.3)	41 (17.7) ^ab,ac,ad^	65 (32.3)	21 (34.4)	81 (42.2)	<0.001

Chi-square tests or Fisher’s exact tests were used to detect the differences in percentages of abnormal blood pressure among the four groups, and post hoc tests were performed with z-tests. ^ab^, *p* < 0.05 for the comparison between the non-obese with non-severe OSA group and the non-obese with severe OSA group. ^ac^, *p* < 0.05 for the comparison between the non-obese with non-severe OSA group and the obese with non-severe OSA group. ^ad^, *p* < 0.05 for the comparison between the non-obese with non-severe OSA group and the obese with severe OSA group. p^1^, overall comparison between the four groups. Abbreviations: OSA, obstructive sleep apnea; hyper-SBP, hyper-systolic blood pressure; hyper-DBP, hyper-diastolic blood pressure.

**Table 4:** Logistic regression models of selected factors and abnormal blood pressure.

	Hyper-SBP		Hyper-DBP		Hypertension	
	OR (95%CI)	*p*	OR (95%CI)	*p*	OR (95%CI)	*p*
Gender	0.647 (0.330, 1.271)	0.206	0.843 (0.439, 1.621)	0.609	0.747 (0.420, 1.331)	0.322
Age	1.031 (1.014, 1.049)	<0.001	1.006 (0.990, 1.023)	0.436	1.022 (1.007, 1.037)	0.004
Smoking	1.061 (0.692, 1.628)	0.785	1.297 (0.856, 1.965)	0.219	1.162 (0.792, 1.703)	0.443
Drinking	1.299 (0.774, 2.181)	0.322	0.929 (0.547, 1.576)	0.784	1.077 (0.668, 1.737)	0.762
Glucose	1.284 (1.105, 1.491)	0.001	1.198 (1.038, 1.382)	0.013	1.250 (1.083, 1.441)	0.002
Obesity	2.695 (1.335, 5.440)	0.006	2.023 (0.949, 4.313)	0.068	2.365 (1.233, 4.535)	0.010
Severe OSA	1.596 (0.936, 2.721)	0.086	2.702 (1.585, 4.607)	<0.001	2.285 (1.438, 3.631)	<0.001
Severe OSA*Obesity	0.670 (0.288, 1.558)	0.352	0.694 (0.290, 1.659)	0.412	0.636 (0.294, 1.373)	0.249

Abbreviations: OSA, obstructive sleep apnea; SBP, systolic blood pressure; DBP, diastolic blood pressure; OR, odds ratio; CI, confidence interval.

## Data Availability

All authors had access to and take responsibility for the data and analyses. Relevant data are available for research upon reasonable request (yunyanxiaxyy@163.com). Data will be saved for 5 years.
